# Application of S-1 Bifactor Model to Evaluate the Structural Validity of TMMS-24

**DOI:** 10.3390/ijerph18147427

**Published:** 2021-07-12

**Authors:** Daniel Ondé, Jesús M. Alvarado, Santiago Sastre, Carolina M. Azañedo

**Affiliations:** 1 Department of Psychobiology & Behavioral Sciences Methods, Complutense University of Madrid, 28223 Madrid, Spain; jmalvara@ucm.es; 2 Department of Psychology, Villanueva University, 28034 Madrid, Spain; ssastre@villanueva.edu (S.S.); cmartin@villanueva.edu (C.M.A.)

**Keywords:** Trait Meta-Mood Scale, perceived emotional intelligence, bifactor, bifactor S-1, explained common variance, percentage of correlations uncontaminated, omega hierarchical

## Abstract

(1) Background: Recent studies have shown that the internal structure of TMMS-24 can be conceptualized as a bifactor. However, these studies, based exclusively on the evaluation of the fit of the model, fail to show the existence of a general factor of strong emotional intelligence and have neglected the evaluation of the specific factors of attention, clarity and repair. The main goal of this work is to evaluate the degree of determination and reliability of the specific factors of TMMS-24 using a bifactor S-1 model. (2) Methods: We administered TMMS-24 to a sample of 384 students from middle and high schools (58.1% girls; mean age = 15.5; SD = 1.8). (3) Results: The specific TMMS-24 factors are better determined and present a higher internal consistency than the general factor. Furthermore, the bifactor S-1 model shows the existence of a hierarchical relationship between the attention factor and the clarity and repair factors. The S-1 bifactor model is the only one that was shown to be invariant as a function of the sex of the participants. (4) Conclusions: The S-1 bifactor model has proven to be a promising tool for capturing the structural complexity of TMMS-24. Its application indicates that it is not advisable to use the sum score of the items, since it would be contaminated by the attention factor. In addition, this score would not be invariant either, that is, comparisons by sex would be invalid.

## 1. Introduction

Recently, several confirmatory factor analysis (CFA) studies have focused on the evaluation of the psychometric properties of the Trait Meta-Mood Scale 24 (TMMS-24) [1] in different Hispanic populations. In most of these studies, the authors have opted to test the original theoretical structure of three correlated factors: attention, clarity and repair. On the other hand, Blasco-Belled et al. [2] and Tejada-Gallardo et al. [3] have proposed a bifactor structure as a better approach to conceptualizing TMMS-24 measurement.

Using a bifactor to evaluate the dimensionality of TMMS-24 is a useful tool, since it allows one to separate the variance that is common to all the items from the variance due to each factor. However, a common characteristic of these studies is that the validity of the internal structure of TMMS-24 has been based mainly on empirical fit measures (i.e., goodness-of-fit indices or GoF), showing that the bifactor model fits better than the three-factor model. In our opinion, this practice reflects some important inconsistencies that should be reviewed.

First, the bifactor model has been applied based on the hypothesis that there is a general emotional intelligence factor that can explain the ability to reason about emotions, while specific emotional factors (attention, clarity and repair) can describe independent abilities. Nevertheless, the better fit of the bifactor model could be a symptom of overparameterization and should not be taken as evidence in favor of the existence of a theoretical general factor [4].

Second, to determine if the underlying structure of the data is more or less multidimensional, the focus should be on the degree of relationship that exists between the three TMMS-24 factors, clearly identifying what part of the common variance is due to general aspects of the measure and what part is due to specific aspects. If the common variance for all the items, identified in the model as a general factor, is strong enough, the measure can be considered as essentially unidimensional [5,6], making it appropriate to use the latent factor score, or the total score (if the tau-equivalence assumption holds) to scale individuals on the evaluated emotional intelligence construct.

The way of understanding the TMMS-24 factor structure has important consequences that transcend the question of the dimensionality of the data, since they imply how the scale scores should be used and interpreted (i.e., as a single total score or as a score for each factor). The GoF do not allow one to assess the degree of multidimensionality or unidimensionality that underlies the data [6], making it necessary to use other indices to assess the strength of the general and specific factors, as well as their degree of internal consistency [5,6,7,8,9] and these issues have been neglected to date.

In the present work, we have analyzed different measurement models in a Spanish adolescent sample. Beyond evaluating the fit of the models, the main goal is to show the utility of the classical bifactor model and bifactor S-1 as a tool to evaluate the strength of the TMMS-24 general factor versus the three specific factors to establish the extent to which the measure can be considered multidimensional or essentially unidimensional, as well as its internal consistency. In addition, an application of a bifactor S-1 model is presented [10,11] in which the presence of a general factor (attention) and two correlated specific factors (clarity, repair) are combined, in an attempt to reconcile both approaches (the presence of general common variance to all items and a theoretical model of three factors). The relevance and suitability of each CFA model are discussed, assessing the current state of the validity of TMMS-24.

### 1.1. Review of Studies Evaluating the Internal Structure of TMMS-24

Recent studies have shown the adequacy of the three correlated factor model [12,13], in line with the theoretical model that served as the basis for the construction of the original scale [14] and consistently with the results shown by other studies [15,16,17,18,19,20]. On the other hand, Blasco-Belled et al. [2] and Tejada-Gallardo et al. [3] have evaluated the internal structure of the scale using a bifactor model, with a general *e*(motional) factor or *e*-factor and three specific factors (attention, clarity and repair). Both studies have used samples of adolescents (students between the ages of 13 and 17, approximately) and samples of adults.

Concerning the evidence of the three-factor structure of TMMS-24, Galdona et al. [12] and Gómez-Núñez et al. [13] identified some items with overlapping content. In both studies, the model fit measures that supported the three-factor model were only adequate when the model was re-specified with the correlation between the errors of these items. Other types of re-specifications were conducted in different samples, such as eliminating item 23 [19] or items 5 and 23 [17] due to low factor loading. Anew, the re-specifications of the original three-factor model allowed researchers to obtain GoF values considered to be good or acceptable. In other studies, model re-specifications were not necessary to achieve adequate model fit [15,18], although some problems with item 23 were reported (low factor loading and a differential item functioning or DIF). Finally, Blasco-Belled et al. [2] and Tejada-Gallardo et al. [3] showed a lack of fit for the three-factor model.

Although the number of re-specifications of the model is not too large, what most of the revised studies reflect is a difficulty in stably showing empirical consistency in different samples. This situation is common in traditional applications of CFA models, such as correlated factors models, due to the restrictiveness imposed by the analytical approach [7,21], in which items are required to load on only one factor and all error correlations (i.e., content overlapping) are constrained to be zero. When the data reflect some item complexity, as in the applied scenario we have revised, the CFA parameter constraints can make it hard to obtain a good model fit, even with cross-loadings and correlations between errors that are not too high. In the TMMS-24 evaluation context, this result is used as an argument favorable to the bifactor model [2,3] since the bifactor model fits better than the three-factor model. The bifactor model is a less restrictive model than the traditional CFA model. In a bifactor model, the general factor reflects a good part of the complexity of the items, minimizing the residuals obtained after fitting the model and, therefore, improving the model fit measures.

On the other hand, beyond the better fit of the model the *e*-factor only has applied interest if it is assumed that the measure is essentially unidimensional (i.e., the *e*-factor must be stronger than the specific factors). An essentially unidimensional measure allows one to consider the use of the total scores of the scale, albeit in the presence of a certain degree of multidimensionality (or factorial complexity of the items), reflected by the different specific factors. Several indices exist to assess the degree of strength of the general factor in a bifactor model, the most recently recommended being explained common variance (ECV), in combination with the percentage of correlations uncontaminated (PUC) and hierarchical omega coefficient (ω*_H_*). To consider a measure as essentially unidimensional, some authors have recommended obtaining ECV values > 0.60 and ω*_H_* > 0.70 for PUC values < 0.80 [6] and ECV values > 0.70 for PUC > 0.70 [8,9].

In none of the studies that use the bifactor model are the recommendations on essential unidimensionality fulfilled. In Blasco-Belled et al. [2], ECV, PUC and ω*_H_* values are not shown. The estimated factor loadings for the bifactor model are included in the Appendix B and we have calculated these values as follows: ECV = 0.348, PUC = 0.696 and ω*_H_* = 0.566. In Tejada-Gallardo et al. [3], the ECV was 0.45 (we estimate ω*_H_* = 0.638). Another way to assess the strength of the general factor is by inspecting the estimated factor loadings, since all the items should reflect similar values [11]. In Blasco-Belled et al. [2], the factor loadings in the general factor ranged between 0.010 and 0.600, while in Tejada-Gallardo et al. [3], they ranged from 0.240 to 0.650. However, it may be premature to reject the *e*-factor hypothesis in this evaluation context.

### 1.2. Bifactor and Bifactor S-1 Models in the TMMS-24 Evaluation Context

**Figure 1:**
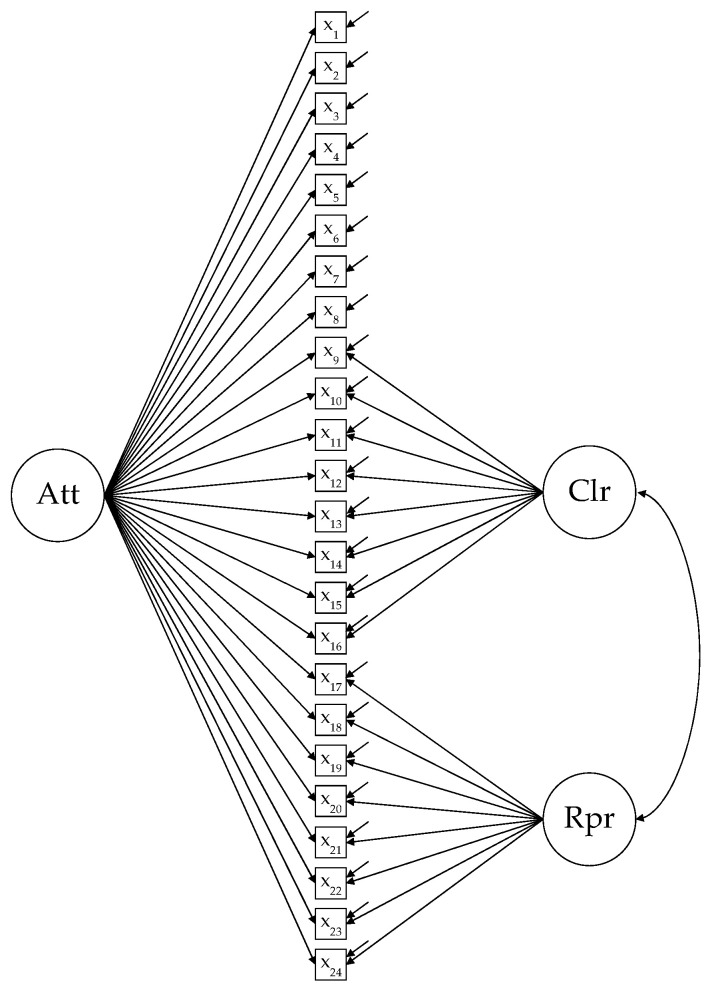
Path-diagram of bifactor S-1_CR_ model. Att = attention; Clr = clarity; Rpr = repair.

The proposal that the true construct is a bifactor model (e.g., like the traditional intelligence concept) should not be confused with the bifactor model as a methodological tool [4]. The classical bifactor model can provide a solid foundation for conceptualizing psychological constructs while allowing the psychometric properties of construct measures to be evaluated. The application of the bifactor model in the evaluation of TMMS-24 has shown a good model fit, although there is insufficient evidence to date for an essentially unidimensional *e*-factor. There are several aspects of the utility of the bifactor model is due to several reasons. The decomposition of the variance of the responses of each item into two direct effects (i.e., general and specific factor) allows one to assessing its factorial complexity in a more precise and interpretable way than the modification indices (MIs) or univariate Lagrange multipliers of the traditional correlated-factors CFA model. Although the MIs are shown simultaneously in a typical CFA output, each MI reflects the difference in chi-square by adding a single parameter to the model (for example, a correlation between the error terms of two items). The problem is that the inclusion of some parameters can produce significant changes in the model, which makes it difficult to obtain an adequate overview. In contrast, in a bifactor model, the researcher can evaluate the complexity of the items simultaneously, assessing whether the responses to each item are due more to the variability common to all items, to the specific variability of a set of items, to both sources of variation, or none of them. More important is that the bifactor model allows one to assess the factorial validity of the subscales or specific factors scores, controlling for the variance due to the general factor (i.e., shared by all the items on the scale), an aspect that has not received attention to date in TMMS-24 evaluation studies. When using the bifactor model, in addition to using ECV and PUC to assess the degree of unidimensionality of the scale, it is recommended to use ω*_H_* [7,22,23] to assess the degree to which the scores of the scale are interpretable as a single measure. This is because a scale can be considered unidimensional and, at the same time, produce scores that primarily reflect noise rather than signal (i.e., lack of reliability) [5]. On the other hand, as Fernández-Berrocal and Extremera [24] point out, there is a way of understanding the structural relationship between the three TMMS-24 factors that implies mediation effects. More specifically, it has been observed that the clarity factor can mediate the relationship between attention and repair [25]. These results suggest that, first, adequate emotional clarity is not possible without a minimum level of attention to feelings and that, second, the capacity to repair our emotions is insufficient unless there is some clarity of emotions and moods [24]. In our opinion, this mediation effect reflects a hierarchical structural relationship between the attention factor and the clarity and repair factors, an aspect that has not received enough focus to date. This hierarchical relationship between factors can be evaluated at the item level using an S-1 bifactor model [10,11]. The S-1 bifactor model uses one of the specific factors as a general reference factor and analyzes the rest of the specific factors or S(pecific)-1 factors, controlled by the common variance of the general reference factor. For TMMS-24, it seems reasonable to use attention as a general factor and clarity and repair as S-1 specific factors. Thus, the S-1 model makes it possible to assess the need to set the items of the attention factor (as a general factor) to factorially determine the specific factors (in this case, clarity and repair), following the hierarchical structure proposed from construct theory. In addition, several of the reviewed studies show a correlation between the patterns of factors where there is a higher relationship between clarity and repair [12,13,17,20]. The S-1 bifactor model with attention as a general reference factor allows us to study the clarity–repair relationship (S-1_CR_ model) more precisely due to the statistical control of the general common variance, since these models allow one to estimate this parameter among the S-1 factors (in the bifactor model, all factors are independent). Figure 1 shows the path diagram for the bifactor S-1_CR_ model with attention as a general factor.

Finally, it may also be preferable to test the bifactor or the S-1_CR_ bifactor models to evaluate multigroup invariance, since the three-factor model does not present adequate fit to overcome the least restrictive level of invariance (i.e., configural invariance). For example, Gómez-Núñez et al. [13] have shown evidence of invariance of TMMS-24 by sex and age once the three-factor model has been re-specified.

## 2. Materials and Methods

### 2.1. Participants

A Spanish student sample was obtained by non-probabilistic sampling in two middle- and high-school educational centers in the Madrid metropolitan area (*N* = 384; 58.1% girls; mean age of 15.5 years (SD = 1.8)). The educational centers were selected by convenience and agreed to participate in the study. The principal of each educational center informed the students’ parents about the study and its objectives. All parents signed an informed consent for the participation of the children. The present study protocol was authorized by the Ethics Committee of Villanueva University (Madrid, Spain; Ref. 2020-27).

### 2.2. Procedure

TMMS-24 was applied in the classroom of the participants during a tutorial session. Before responding, the researchers read aloud a set of instructions, which described the type of statements of the instrument and how to answer. Once finished, the students would have explained what emotional intelligence is and how it can positively affect their studies, relationships, etc.

### 2.3. Instruments

The Trait Meta-Mood Scale (TMMS-48) [14] is a widely used self-report instrument for measuring the affective capacity of people in their daily lives over time [24]. This scale focuses on evaluating the beliefs that people have about their moods and emotions (Perceived Emotional Intelligence, PEI) [26] and was initially conceived as a measure composed of three emotional factors: attention (amount of attention paid to one’s emotional states), clarity (understanding one’s emotional states) and repair (ability to regulate one’s emotional states). The TMMS-48 has a total of 48 items, 21 for attention, 15 for clarity and 12 for repair. Individuals are asked to evaluate the degree of agreement with each item using a five-point ordered scale (1 = totally disagree; 5 = totally agree). 

The TMMS-48 was adapted to the Spanish language by Fernández-Berrocal et al. [27]. The Spanish version of the TMMS-48 was shortened by reducing its length to 24 items (TMMS-24), maintaining the original three-factor structure (8 items per factor) [1,27]. It is the most commonly used self-report instrument in Spanish language [13].

### 2.4. Data Analysis

Preliminary analysis was elaborated using the SPSS 25.0 program (IBM Corp., Armonk, NY, USA). Then, we conducted confirmatory factor analysis (CFA) to fit four measurement models: three correlated factors (3FAC), bifactor (BIF), bifactor S-1 without correlation between clarity and repair (S-1) and bifactor S-1 with clarity-repair correlated factors (S-1_CR_). We also conducted a Multigroup-CFA (MG-CFA) across sex (girls and boys). We used the polychoric correlation matrix as input matrix, appropriate when analyzing ordinal variables (i.e., items) [28,29]. For the CFA and MG-CFA, we used the lavaan package for the R program [30] and the estimator Diagonal Weighted Least Squares (DWLS), recommended for analyzing samples with a small to moderate number of observations with ordinal data [31].

For the CFA model fit evaluation, we used the χ^2^ test, the Root Mean Square Error of Approximation (RMSEA), Standardized Root Mean Square Residual (SRMR), Comparative Fit Index (CFI) and Tucker–Lewis Index (TLI). For the MG-CFA, we conducted a single-group CFA (girls and boys) and three invariance CFA models sequentially: configural invariance or equal form (M1), metric/weak invariance or equal-factor loadings (M2) and scalar/strong invariance or equal-intercepts (M3). We used delta parameterization, the most common approach for MG-CFA. When using the delta approach in MG-CFA with ordinal data, the residual variances of the items are not allowed to be parameters of the models, so strict invariance (M4) cannot be tested. Nevertheless, to consider that the scores obtained in the TMMS-24 by girls and boys are comparable, it is not necessary to test the equality of item residual variances [28].

Both ECV and ω values for specific factors can also be obtained (ECV*_S_* and ω*_HS_*, respectively). ECV*_S_* reflects the proportion of common variance that explains a specific factor relative to the variance that all factors explain and ω*_HS_* reflects the proportion of explained variance of the specific factors scores while controlling for the general factor [8,9,32], that is, independently of the general factor. We obtained the ECV, PUC and w values using a Microsoft Excel-based tool [33].

## 3. Results

### 3.1. Preliminary Analysis

The means of the 24 items ranged between 2.6 and 4.6. The lowest standard deviation (SD) was 0.7 and the highest 1.2 (see Appendix A, Table A1). All the asymmetry and kurtosis values were within the ±1 range, except for item 23 (asymmetry = −2.1, kurtosis = 5.3). The asymmetry and kurtosis values are consistent with previous studies [13,18]. Response rates were practically 100%, with a maximum of 5 omissions in item 5.

### 3.2. CFA Model Fit

Table 1 shows the fit obtained for each of the models tested.

We used the following criteria for evaluating model fit: RMSEA < 0.08, SRMR close to 0.8 or below, CFI and TLI > 0.95 [28]. The 3FAC model does not meet the criteria for RMSEA or SRMR. The inspection of the MIs suggested that the specification of several parameters could improve the fit of the 3FAC model (more specifically, factor loadings for item 5 in the clarity and repair factor, factor loadings for items 13 and 22 in the attention factor and correlations between errors for items 14–15, 2–22, 4–22, 4–13 and 5–11). We considered MI values higher than 7.88 (99.5th percentile of the χ^2^ distribution with 1 df [34]). These results are consistent with previous studies [2,3,12,13] and also consistent with previous studies [2,3], the BIF model reflects the better fit (i.e., is the less restrictive model). The S-1_CR_ model obtains a slightly worse fit than the BIF model because it is more restrictive, although it is also a model that reflects adequate empirical consistency. The S-1_CR_ model has a better GoF performance than the S-1 model. Since it is the model most consistent with previous studies (i.e., a higher correlation between clarity and repair) [12,13,17,20], we chose the S-1_CR_ model to evaluate the hypothetical hierarchical relationship between the TMMS-24 factors [24].

### 3.3. Degree of Multidimensionality and Reliability

For the 3FAC model, the correlations between factors are 0.262 for attention–clarity, 0.277 for attention–repair and 0.343 for clarity–repair. For the S-1_CR_ model, the correlation between clarity and repair was 0.289 after controlling for the attention factor variance. These results are consistent with previous studies [13,17]. ECV and PUC was 0.313 and 0.696, respectively. These results are consistent with previous studies [2,3] and indicate no evidence of unidimensionality of the TMMS-24 measure with the available data. However, given this result, it is worth asking about the degree of determination of the specific factors. In this study, the ECV*_S_* values were 0.730 (attention), 0.675 (clarity) and 0.647 (repair). These results are compatible with the original three-factor conceptualization of the meta-mood trait measure proposed by Salovey et al. [14]. The inspection of the estimated factor loadings values (Table A2, Appendix B) is also useful to establish the degree of determination of the evaluated factors. The range of factor loadings were −0.125–0.644 (general factor), 0.520–0.801 (attention), 0.289–0.798 (clarity) and 0.213–0.819 (repair). The factor loadings in the general factor are quite heterogeneous, even showing a negative factor load in item 5 (−0.125). Among the specific factors, the only factor that reflects relatively homogeneous factor loadings is attention. We used ω*_HS_* to estimate the reliability of the general and the three specific factors of the BIF model and the values were ω*_H_* = 0.521, ω*_HAttention_* = 0.709, ω*_HClarity_* = 0.605 and ω*_HRepair_* = 0.533.

The results show that the general factor is poorly determined (i.e., low ECV and ω*_H_* values). On the other hand and following the recommendations of Reise et al. [6], the only specific factor that presents a sufficient level of ECV and ω*_HS_* is attention (ECV > 0.60 and ω*_HS_* > 0.70). We also calculated the ECV, ω*_H_* and ω*_HS_* values for the S-1_CR_ model. The ECV for attention, clarity and repair was 0.415 (PUC = 0.797), 0.907 and 0.895, respectively. The ω*_H_* for attention was 0.540 and the ω*_HS_* for clarity and repair was 0.828 and 0.779, respectively. These results reflect high consistency of the clarity and repair factors by controlling for the attention factor. The estimated factor loadings in the S-1 model range between 0.019 and 0.843 (attention), 0.484 and 0.808 (clarity) and 0.379 and 0.849 (repair).

### 3.4. Multigroup-CFA Analysis Based on Sex

We first tested whether the 3FAC, BIC and S-1_CR_ models were acceptable in single-group analysis and configural invariance (M1) tests. The GoF values for the 3FAC model reflected a poor fit for single-group analysis and M1, so it did not make sense to continue evaluating the rest of the nested models. The BIF and S-1_CR_ models obtained adequate GoF values for single-group analysis and M1. For both BIF and S-1_CR_ models, we tested equivalence between nested invariance models M1, M2 and M3 by a nonsignificant probability level of χ^2^ (*p*-value > 0.05), a recommended approach to test MG-CFA invariance models with ordinal data, two groups, moderate-low sample size and models with 2–4 factors [35,36]. For the BIF model, there was a significant difference between M2-M1 χ^2^ tests, while the S-1_CR_ model reflected nonsignificant differences χ^2^ between M1-M2 and M3-M2. Consequently, only the S-1_CR_ model shows the property of invariance, a requirement to compare the scores of girls and boys (see Table 2). 

## 4. Discussion

To date, most of the studies that analyze the internal structure of TMMS-24 justify the adequacy of one model or another based on the GoF indices. Studies that have fitted the traditional CFA model of three correlated factors do not always reflect a good fit. This situation has been used as an argument in favor of the bifactor model due to its better performance in the GoFs. However, as with any other CFA model [37,38], a good-fitted bifactor model does not necessarily imply theoretical consistency [4,5]. On the other hand, the adequation of model fit indices is not valid to answer the question of whether TMMS-24 can be understood as a multidimensional or unidimensional factor structure, which implies determining how the scale scores should be used (single total score, three subscale scores and factor scores by dimension).

The main goal of this work is to show the utility of the bifactor model as a tool for evaluating the extent to which the factor structure of TMMS-24 can be considered multidimensional or unidimensional enough. To date, the bifactor model has been used to evaluate TMMS-24 based on the hypothesis that there is an underlying *e*-factor [2,3]. However, although the bifactor model reflects a better GoF performance compared with the traditional CFA model, the results obtained do not allow us to assume that the TMMS-24 measure is essentially unidimensional (i.e., low ECV and ω*_H_* values). We re-analyzed the bifactor model in a Spanish adolescent sample, obtaining the same results. The lack of unidimensionality shown by current bifactor results raises the need to assess the consistency of specific factors, an aspect of the bifactor model neglected in previous studies. Our results show that the specific factors are better determined than the general factor, indicating the multidimensional nature of the TMMS-24 measurement from an evaluation of the internal structure point of view. Does this mean that the most theoretically correct measurement model is the original model of three correlated factors? The results of the bifactor model support the three-factor hypothesis, although it is conceivable that the nature of the measure may be somewhat more complex. The S-1_CR_ bifactor model has allowed us to test a model that reflects a hierarchical relationship between the attention factor and the clarity and repair factors. This way of understanding the internal structure of the TMMS-24 is of interest, since a minimum of emotional attention is required for individuals to perceive some emotional clarity and for emotional repair to occur, individuals must perceive their emotions with a minimum of clarity [24]. The S-1_CR_ bifactor model fits slightly worse than the bifactor model, as it is more restrictive, although it has shown an adequate level of empirical consistency. More importantly, the results of the bifactor S-1_CR_ show that clarity and repair are two factors adequately determined after controlling for the attention general reference factor. In addition, bifactor S-1_CR_ is the only one of the evaluated models that obtained evidence of invariance by sex (girls and boys). However, it should be noted that ECV, PUC and ω are not informative for decisions about fitting different multidimensional models to the data [5,39]. The choice of which multidimensional model is optimal must be guided by substantive theory. The bifactor S-1_CR_ model is a promising new approach to assessing TMMS-24, although additional research is required for establishing the mediation/hierarchy hypothesis between attention, clarity and repair. In this sense, the bifactor S-1_CR_ model has shown that the clarity and repair factor scores can be used with an adequate degree of internal consistency (i.e., ω*_HS_* > 0.70), which will undoubtedly be of interest to different applied researchers that want to analyze the degree of relationship between TMMS-24 and other variables. It should be noted that to use specific factor scores accurately (i.e., controlling by attention factor), we recommend calculating factor scores rather than sum scores.

Finally, the type of restrictions imposed by the traditional CFA model implies assuming that the underlying measure is factorially simple, which conflicts with the results obtained in different samples. The bifactor model is less restrictive, allowing the degree of factor complexity of the items to be analyzed from a more direct and interpretable perspective than the questionable use of MIs [28] of the traditional CFA model. MIs are helpful for detecting factor complexity, although it can be difficult to interpret in applied contexts, since their significance frequently changes when the model is re-specified by including other parameters. The bifactor model makes it possible to simultaneously assess whether the variance of each item is mainly due to the all-item common variance, some specific factor, or both sources of variation. In our study, some items with low factor loadings in the general factor (items 5, 6 and 11) and some others with low factor loadings in the specific factors (item 13 in clarity, items 22 and 23 in repair) arise. This result coincides with that suggested by the MI analysis, although it is easier to interpret, making it more convenient to identify troublesome items.

## 5. Limitations

Our results were obtained from a sample of middle- and high-school students. Therefore, the ability to generalize our results is limited, requiring new studies with adult populations that also study the strength of specific factors and their potential hierarchical relationship. In this sense, TMMS-24 was validated with a university population [24], so the difference in age and educational level could act as confounding variables to take into account in future research.

Furthermore and concerning the characteristics of the analyzed sample, even though the results reflect an evident lack of unidimensionality, we do not want to suggest that the *e*-factor hypothesis should be rejected. A good indicator of a general factor is the presence of strongly related specific factors. In Gómez-Núñez et al. [13] and Martín-Albo et al. [17], the correlations between factors range from 0.26 to 0.48 and from 0.14 to 0.43, respectively. These correlations are not very high, so a strong general factor is not expected. On the other hand, Galdona et al. [12] and Valdivia et al. [20] reported higher factor correlations (0.39–0.54 and 0.35–0.63, respectively). However, we do not know whether they are high enough to support essentially unidimensional evidence, since bifactor was not used in these studies. Additional research is needed to assess the potential unidimensionality of TMMS-24. In this sense, empirical evidence of the strength of the general factor could emerge in some subsamples’ a/o application contexts (age-subsamples of specific organizational contexts, clinical subsamples, etc.).

Concerning future studies, we encourage researchers to use the bifactor S-1 model in research on the hierarchical nature of emotional intelligence. In the case of TMMS-24, we have opted for a model (S-1_CR_) that has theoretical support behind it. However, it should be noted that the models proposed in this work do not exhaust all the possibilities of analysis. Of course, any researcher can test other models from the same theoretical assumptions or others, with or without a hierarchical relationship, or by specifying other factors as general reference factors in the case of S-1 models. Testing other models is beyond the scope of this work, since we intended to show the performance of some specific models that had not been considered to date in this area.

Finally, this study should be complemented with an analysis of the relationships between the different proposed factor solutions and other variables of the nomological network of emotional intelligence.

## 6. Conclusions

Currently, there are few studies that use a bifactor model to evaluate TMMS-24. We recommend its application as a methodological tool. Given the factor complexity that some items have shown in applied contexts, the bifactor model is a more helpful evaluation tool than the traditional three-factor CFA model, although we recommend that researchers routinely use and compare both models.

In addition to factor complexity at the item level, TMMS-24 could reflect factor complexity at the structural level. The S-1_CR_ bifactor model is a promising tool to evaluate this type of complexity because it allows the specification of hierarchical relationships between the different factors. Furthermore, thanks to the statistical control exerted on the attention factor, bifactor S-1_CR_ may help obtain precise factor scores in the clarity and repair factors, for which we recommend its application in evidence-based studies aimed toward criterion validity of TMMS-24. Its application indicates that it is not advisable to use the sum score of the items, since it would be contaminated by the attention factor. In addition, this score would not be invariant either, that is, comparisons by sex would be invalid.

## Figures and Tables

**Table 1:** Goodness-of-fit indices for the 3FAC, BIF, S-1 and S-1_CR_ models.

Model	χ^2^ (df)	CFI	TLI	RMSEA (90% CI)	SRMR
3FAC	1006.7 * (249)	0.957	0.952	0.091 (0.086–0.097)	0.087
BIF	440.2 * (228)	0.988	0.985	0.051 (0.043–0.058)	0.056
S-1	919.2 * (236)	0.961	0.954	0.089 (0.083–0.095)	0.085
S-1_CR_	588.8 * (235)	0.980	0.976	0.064 (0.058–0.071)	0.066

* *p* < 0.05.

**Table 2:** Goodness-of-fit indices for the single-group analysis and MG-CFA for BIF and S-1_CR_ models based on sex (girls and boys).

		χ^2^	df	CFI	TLI	RMSEA (90% CI)	SRMR	Model Comparison	Δχ^2^ (Δdf, *p*-Value)	ΔCFI
BIF	Girls	292.2	228	0.994	0.993	0.036 (0.022–0.048)	0.059			
	Boys	286.9	228	0.991	0.989	0.042 (0.024–0.056)	0.073			
	M1	579.0	456	0.993	0.991	0.039 (0.028–0.048)	0.065			
	M2	824.1	500	0.981	0.979	0.060 (0.052–0.067)	0.076	M1–M2	79.0 (44, <0.001)	0.012
S-1_CR_	Girls	470.1	235	0.978	0.975	0.068 (0.059–0.077)	0.076			
	Boys	333.2	235	0.985	0.982	0.053 (0.039–0.066)	0.078			
	M1	803.4	470	0.981	0.978	0.063 (0.055–0.070)	0.077			
	M2	965.2	507	0.974	0.971	0.071 (0.064–0.077)	0.084	M1–M2	49.8 (37, 0.078)	0.007
	M3	1013.5	576	0.975	0.976	0.065 (0.058–0.071)	0.082	M3–M2	70.2 (69, 0.437)	−0.001

*Notes*. M1 = configural invariance; M2 = metric invariance; M3 = scalar invariance; χ^2^ = Chi-square; df = degrees of freedom; CFI = Comparative Fit Index; TLI = Tucker–Lewis Index; RMSEA = Root Mean Square of Approximation; SRMR = Standardized Root Mean Square Residual; Δχ^2^ = χ^2^ difference model comparison test; Δdf = difference between degrees of freedom; ΔCFI = CFI difference model comparison.

## Data Availability

doi:10.6084/m9.figshare.14954892.v1.

## Appendix A

**Table A1:** Descriptive statistics of 24 items of TMMS-24.

Items	M (SD)	Median	Skewness (Kurtosis)
Attention1	3.7 (1.0)	4	−0.31 (−0.82)
Attention2	3.5 (1.1)	4	−0.17 (−1.04)
Attention3	3.1 (1.2)	3	0.03 (−0.89)
Attention4	3.7 (1.1)	4	−0.48 (−0.74)
Attention5	3.3 (1.2)	3	−0.22 (−0.99)
Attention6	2.8 (1.2)	3	0.23 (−0.80)
Attention7	3.1 (1.2)	3	0.07 (−0.88)
Attention8	3.2 (1.1)	3	−0.03 (−0.84)
Clarity1	3.1 (1.3)	3	−0.06 (−1.06)
Clarity2	3.0 (1.2)	3	0.00 (−0.89)
Clarity3	3.2 (1.2)	3	−0.11 (−0.96)
Clarity4	3.5 (1.0)	4	−0.33 (−0.56)
Clarity5	3.6 (1.0)	4	−0.38 (−0.40)
Clarity6	2.7 (1.2)	3	0.26 (−0.80)
Clarity7	3.1 (1.0)	3	−0.14 (−0.66)
Clarity8	3.3 (1.1)	3	−0.17 (−0.71)
Repair1	3.3 (1.3)	3	−0.28 (−0.94)
Repair2	3.2 (1.2)	3	−0.16 (−0.97)
Repair3	2.6 (1.3)	2	0.42 (−0.87)
Repair4	3.2 (1.2)	3	−0.12 (−0.98)
Repair5	3.2 (1.2)	3	−0.17 (−0.84)
Repair6	3.6 (1.1)	4	−0.54 (−0.34)
Repair7	4.6 (0.7)	5	−2.12 (5.27)
Repair8	3.2 (1.1)	3	−0.03 (−0.82)

## Appendix B

**Table A2:** Estimated factor loadings (standards errors in parentheses) for 3FAC, BIF, and S-1_CR_ models.

Items	3FAC Model			BIF Model ^1^				S-1_CR_ Model ^2^		
	Attention	Clarity	Repair	G	Attention	Clarity	Repair	Attention	Clarity	Repair
Attention1	0.667 (0.034)			0.409 (0.058)	0.520 (0.048)			0.671 (0.033)		
Attention2	0.816 (0.021)			0.514 (0.055)	0.623 (0.043)			0.814 (0.021)		
Attention3	0.773 (0.024)			0.413 (0.059)	0.646 (0.040)			0.771 (0.024)		
Attention4	0.720 (0.030)			0.494 (0.060)	0.520 (0.050)			0.724 (0.029)		
Attention5	0.393 (0.043)			−0.125 (0.064)	0.625 (0.046)			0.400 (0.043)		
Attention6	0.652 (0.032)			0.191 (0.067)	0.671 (0.041)			0.648 (0.032)		
Attention7	0.829 (0.021)			0.316 (0.063)	0.801 (0.032)			0.826 (0.021)		
Attention8	0.846 (0.021)			0.473 (0.059)	0.693 (0.042)			0.843 (0.021)		
Clarity1		0.726 (0.027)		0.379 (0.063)		0.623 (0.045)		0.154 (0.052)	0.714 (0.030)	
Clarity2		0.769 (0.023)		0.328 (0.065)		0.717 (0.038)		0.127 (0.052)	0.771 (0.025)	
Clarity3		0.752 (0.025)		0.228 (0.070)		0.798 (0.033)		0.019 (0.054)	0.808 (0.021)	
Clarity4		0.574 (0.036)		0.413 (0.060)		0.388 (0.055)		0.254 (0.049)	0.500 (0.039)	
Clarity5		0.634 (0.037)		0.610 (0.063)		0.289 (0.064)		0.410 (0.046)	0.484 (0.036)	
Clarity6		0.769 (0.025)		0.331 (0.066)		0.722 (0.040)		0.103 (0.052)	0.786 (0.025)	
Clarity7		0.714 (0.028)		0.400 (0.063)		0.594 (0.040)		0.181 (0.050)	0.691 (0.026)	
Clarity8		0.740 (0.027)		0.532 (0.060)		0.513 (0.049)		0.269 (0.048)	0.680 (0.031)	
Repair1			0.706 (0.028)	0.354 (0.064)			0.626 (0.042)	0.145 (0.051)		0.703 (0.030)
Repair2			0.843 (0.019)	0.339 (0.063)			0.819 (0.031)	0.149 (0.049)		0.849 (0.020)
Repair3			0.684 (0.032)	0.348 (0.063)			0.591 (0.040)	0.181 (0.052)		0.660 (0.031)
Repair4			0.830 (0.020)	0.360 (0.065)			0.783 (0.033)	0.127 (0.050)		0.849 (0.018)
Repair5			0.476 (0.045)	0.306 (0.063)			0.355 (0.052)	0.067 (0.053)		0.500 (0.040)
Repair6			0.647 (0.038)	0.640 (0.054)			0.232 (0.069)	0.411 (0.045)		0.467 (0.045)
Repair7			0.483 (0.060)	0.441 (0.075)			0.213 (0.071)	0.268 (0.061)		0.379 (0.061)
Repair8			0.536 (0.042)	0.351 (0.067)			0.399 (0.055)	0.203 (0.051)		0.487 (0.040)

^1^ ECV = 0.313 (PUC = 0.696), ECV*_Attention_* = 0.730, ECV*_Clarity_* = 0.675, ECV*_Repair_* = 0.647, ω*_H_* = 0.521, ω*_HAttention_* = 0.709, ω*_HClarity_* = 0.605, ω*_HRepair_* = 0.533. ^2^ ECV*_Attention_* = 0.540 (PUC = 0.797), ECV*_Clarity_* = 0.907, ECV*_Repair_* = 0.895, ω*_HAttention_* = 0.540, ω*_HClarity_* = 0.828, ω*_HRepair_* = 0.779.
